# Effectiveness of booster strategies to promote physical activity maintenance: a systematic review and meta-analysis

**DOI:** 10.1186/s12966-025-01844-2

**Published:** 2025-11-05

**Authors:** Giampiero Tarantino, Nikos Ntoumanis, Ross Neville, Chiara Cimenti, Anne Poder Petersen, Kristina Pfeffer, Alexandre Mazéas, Malte Nejst Larsen, Peter Krustrup, Cecilie Thøgersen-Ntoumani

**Affiliations:** 1 Department of Sport Science and Clinical Biomechanics, Sport and Health Sciences Cluster (SHSC), University of Southern Denmark, Campusvej 55, Odense, Denmark; 2 DRIVEN, Danish Centre for Motivation and Behaviour Science, Odense, Denmark; 3 Danish Institute for Advanced Study (DIAS), University of Southern Denmark, Odense, Denmark; 4 School of Sport, Exercise, and Rehabilitation Sciences, University of Birmingham, Birmingham, UK; 5 School of Public Health, Physiotherapy & Sports Science, University College Dublin, Dublin, Republic of Ireland; 6 Department of Clinical Medicine, Aarhus University, Nordre Ringgade 1, Aarhus, Denmark; 7 Department of Respiratory Diseases and Allergy, Aarhus University Hospital, Aarhus, Denmark

**Keywords:** Boosters, Physical activity, Moderate-to-vigorous physical activity, Behaviour change, Physical activity maintenance, Intervention, Follow-up prompts

## Abstract

**Background:**

Physical activity (PA) is essential for physical and mental health, yet sustaining long-term PA engagement remains a challenge. Booster strategies—follow-up contacts delivered after the end of interventions—have been proposed as a strategy to support PA maintenance, but their effectiveness remains unclear. The primary objective of this systematic review and meta-analysis was to classify the boosters used in PA interventions depending on their type and number. The secondary objective was to explore the efficacy of boosters in supporting participants’ PA maintenance.

**Methods:**

A systematic search was conducted across seven databases, up to February 2025. Randomised controlled trials were included if they incorporated boosters and reported PA outcomes. Risk of bias was assessed using the RoB 2 tool. Meta-analysis examined changes in moderate-to-vigorous physical activity (MVPA) from baseline to the last available follow-up, and moderation analysis explored the effects of booster type, number of boosters administered, and follow-up duration on changes in MVPA. Studies not suitable for meta-analysis were synthesised narratively.

**Results:**

Forty studies were included in the systematic review. The most common types of boosters used were phone calls and text messages, which were employed either alone or in combination with other types. 16 studies provided data for inclusion in the meta-analysis. There was conclusive evidence that including a booster in the intervention led to sustained increases in PA levels at follow-up. The estimated added effect of the booster over the intervention alone was a 6% increase. There was also conclusive evidence of increased MVPA for interventions with more boosters, and interventions that used remote and mixed-format delivery (vs in-person only) boosters. Finally, results showed conclusive evidence of increased MVPA for interventions that assessed MVPA using self-reported measures.

**Conclusions:**

Our findings suggest a trend indicating that boosters may support the maintenance of PA. Higher number of boosters and delivery through remote or mixed formats showed promising trends. Future research should also explore optimal booster numbers and formats to clarify their role in sustaining PA.

**Registration:**

PROSPERO (CRD42024510018); Protocol also available on Open Science Framework (OSF): https://osf.io/6abkw/?view_only=915375148520427db3dca76d2c32934d.

**Supplementary Information:**

The online version contains supplementary material available at 10.1186/s12966-025-01844-2.

## Introduction

Considering the low cost associated with physical activity (PA) [1] and the wide range of benefits that it can offer (such as preventing the risk of developing cardiovascular disease, diabetes mellitus, osteoporosis, some forms of cancer, and mental health disorders) [2–4], numerous interventions have been implemented over the recent decades to promote PA levels across various contexts [5–8]. Meta-analyses reported a wide range of improvements, from moderate to large, across diverse populations and settings [9–14]. However, regardless of age groups and contextual settings, long-term maintenance of PA levels is rarely achieved, with treatment washout commonly observed [15, 16].

Research has shown that maintaining long-term adherence to PA interventions is essential to achieving health outcomes [15]. One strategy that has been used to support long-term PA behaviour change is the employment of follow-up support strategies implemented after the end of interventions. A variety of such strategies have been used, such as workshops, printed material, newsletters, or phone-based reminders [17–20]. A systematic review of maintenance of behaviour change following PA and dietary interventions found that interventions were more likely to achieve maintenance if they included follow-up prompts, such as brief contact with participants after the delivery of the main intervention [21]. Such follow-up strategies– thereinafter referred to as *boosters* – have also been shown to be effective in self-management interventions for chronic musculoskeletal pain [22]. However, the evidence pertaining to the effects of booster strategies on PA maintenance has not been synthesised and compared to interventions without boosters. This meta-analysis aims to fill this gap.

### Definition of booster

The term booster has been employed in diverse areas within health research. For example, the ‘booster sessions’ terminology has been widely used in research focusing on mental health [23, 24], knee osteoarthritis rehabilitation [25–28], back pain management [29], and cardiac rehabilitation [19, 30]. In these lines of research, scholars have referred to boosters as follow-up maintenance and reinforcement strategies [15, 31], and defined them as ‘brief contacts beyond the main part of the intervention to reinforce previous intervention contents’ [19]. However, despite PA researchers employing the term booster in their work [32, 33], this term has not been defined within the PA domain. In our study we define boosters as ‘any form of support provided to participants after the end of an intervention, in an attempt to maintain the effects of the intervention’.

Researchers have investigated various boosters to keep participants motivated to engage in long-term PA. For example, researchers have suggested the employment of behaviour change techniques (such as goal setting or action planning), in addition to the main intervention, to facilitate behaviour change [34] and increase intrinsic motivation (i.e., autonomous motivation) to PA [35]. Boosters can be delivered in face-to-face formats or remotely via digital technologies (e.g., smartphone applications, web-based platforms, or wearable activity trackers). Indeed, previous research has implemented various technology-assisted methods, such as tailored feedback, goal reminders, and online social support communities, to sustain participant engagement [36, 37]. A meta-analysis found that PA interventions including strategies such as text messaging containing prompts and cues were associated with greater behaviour maintenance [38].

### Definition of PA maintenance

Long-term PA maintenance has been commonly conceptualised with the Transtheoretical Model (TTM), which designates a person as being in the *maintenance* stage once they have sustained regular PA for six consecutive months [39]. The TTM itself does not require that this activity meets the global guideline of ≥ 150 min per week of moderate-to-vigorous PA (MVPA) [40]; thus, the six-month duration has become the standard operational definition in exercise-behaviour research [41]. Because our review focuses on the effectiveness of booster strategies rather than on whether participants achieved guideline-level PA, we defined PA maintenance (thereinafter, *PA sustainment*) as the preservation of the post-intervention PA levels at the last available follow-up assessment.

### Aims of this systematic review and meta-analysis

Despite the potential benefits of boosters as a form of reinforcement to support participants’ PA sustainment, research evidence is lacking on the types of boosters that have been employed and whether these are effective in achieving better maintenance of the PA behaviour change than interventions only. The primary objective of our systematic review was to describe the types of boosters that have been used in PA interventions in relation to their format (such as reminders, phone calls, etc.); the total number of boosters administered; the settings in which they have been administered (such as schools, primary care, communities, etc.); the age groups to which they were administered (such as children, adolescents, and adults); and the target populations (such as clinical and non-clinical). The objectives of our meta-analysis were to (1) explore the effectiveness of booster strategies in supporting participants’ PA sustainment; (2) assess whether the number of boosters administered and (3) the follow-up duration predicted PA sustainment, and (4) investigate which type of boosters were more effective in promoting PA sustainment. We hypothesised that:

H1: Within individual studies, PA interventions that incorporated booster strategies would overall yield better PA sustainment than their comparator arms that did not include boosters.

H2: More boosters would be associated with better PA sustainment levels.

H3: Longer follow-up durations (namely, the time elapsed between the end of the intervention and the last PA measurement) would be associated with better PA sustainment levels.

Finally, as we had no *a prori* hypothesis, exploratory analyses were conducted to explore whether certain types of boosters were associated with better PA sustainment.

## Methods

A systematic review and meta-analysis was conducted in accordance with the Preferred Reporting Items for Systematic Reviews and Meta-Analyses (PRISMA) guidelines [43]. The review was registered in PROSPERO (CRD42024510018) and the protocol was published in Open Science Framework (OSF) [https://osf.io/6abkw/?view_only=915375148520427db3dca76d2c32934d].

### Data sources and search strategy

Seven electronic databases (PubMed, CINAHL, SPORTDiscus, Web of Science, EMBASE, PsycINFO, and Scopus) were searched, from inception up to February 2025. Studies were identified by using all possible combinations of synonyms and spelling of the following group concepts: (a) “booster*”; (b) “physical activity”; and (c) “randomised controlled trial”. MeSH terms and thesaurus headings were also included in the search strategy. The complete search strategy is reported in the study protocol [44] and in the supplementary material. Only articles in English were included. Furthermore, the reference lists of the articles deemed eligible for inclusion were checked for other potentially relevant articles.

### Eligibility criteria

The eligibility criteria were specified a-priori using the PICO (Population, Intervention, Comparator, Outcome) framework [45]. *Population*: human participants of any age, sex, health status, or within any setting were deemed suitable for inclusion. No restrictions were placed on baseline activity level, clinical condition, or demographic characteristics so that subgroup analyses by these factors would be possible. *Intervention*: A structured PA intervention, incorporating one or more booster strategies (e.g., additional counselling session, refresher class, telephone/e-mail prompt) delivered *after* completion of the main intervention phase and intended specifically to maintain PA. The nature, timing, number, and modality of boosters were unrestricted. *Comparator*: at least one concurrent trial arm without a booster, which could be (a) an otherwise identical PA intervention delivered without booster; and/or (b) a minimal-contact/usual-practice control. Trials had to include *both* a booster arm and at least a non-booster comparator arm within the same randomised comparison. *Outcome*: quantitative measures of PA behaviour collected at baseline, post-intervention, and follow-up after receipt of booster. Eligible PA metrics included steps, minutes of moderate-to-vigorous PA (MVPA), metabolic-equivalent minutes, accelerometer counts, etc. Measurement instruments could be devices (accelerometer, pedometer, wearable), and self-report questionnaires. Studies that reported only functional-fitness outcomes (e.g., 30s sit-to-stand) or sedentary behaviour were excluded. *Study design*: Parallel-group randomised controlled trials (RCTs) published as peer-reviewed journal articles in English were deemed suitable for inclusion. Conference abstracts, dissertations, theses, and other grey literature were excluded to ensure robustness in assessing the effectiveness of the interventions.

### Screening process

The articles retrieved by the search strategy were imported into Covidence [46], where duplicates and non RCTs were automatically screened out. The remaining articles were imported into ASReview, a machine learning-based screening tool [47]. ASReview is a free open-source machine learning tool, developed by the Utrecht University, for screening and systematically labelling a large collection of textual data, which has been utilised in several systematic review and meta-analysis [44–46]. Simulation studies have found this tool helpful to identify relevant studies in the title/abstract screening process, improving the efficiency of such a screening process [48]. A complete list of peer-reviewed articles that have used this tool, and papers published by the ASReview team is provided at the following link https://www.zotero.org/groups/4597652/asreview_public/collections/PKKJAVQP/items/G2975U7E/collection. Titles and abstracts were screened according to Option 3 of the Manual for integrating ASReview into a systematic review workflow (https://asreview.nl/blog/seven-ways-to-integrate-asreview/#multiple). Briefly, the title and abstract screening was performed by four reviewers, with two alternating during the screening process until a stopping criterion of 4% was achieved, meaning that after 4% of irrelevant studies (of the total number of studies imported) in a row identified for title/abstract screening, such a screening can finish [48]. In the case of this study, the stopping criterion was set at 730 consecutive non-relevant titles/abstracts. 730 represents the 4% of 18,253 (i.e., the total number of titles/abstracts imported for screening). More details are offered in our study protocol. The full texts of the remaining articles were then imported into Covidence for full text screening. Four researchers (two per article) independently assessed the eligibility of the articles against the inclusion criteria; discrepancies were resolved through discussion, in consultation with the senior authors of the paper.

### Data extraction

We extracted from eligible studies metadata (such as authors’ name, publication year, and country where the study was conducted) and study characteristics (such as participants’ demographics, time points at which the data was collected, intervention content, theoretical framework used, and PA as a primary or secondary outcome). Booster characteristics, comprising of the type(s) and number of booster(s) administered, were also extracted. PA data extracted included: (i) the unit of measurement for PA (e.g., steps, minutes of MVPA, metabolic-equivalent minutes); (ii) the measurement instrument or method (e.g., pedometer, accelerometer, questionnaire); and (iii) the mean ± standard deviation for each study arm at baseline, immediately post-intervention, and at the last available follow-up after booster administration. Corresponding authors were contacted in case of missing information. If no response was provided within two weeks, a follow-up email was sent to the corresponding author. Information regarding the contacting author outcome is reported in Table 1.

**Table 1:** Characteristics of the studies included in this systematic review and meta-analysis

Authors	Year	Country	Setting	Health Condition	Participants			PA measurements			Intervention Content	Theoretical Framework	PA as primary outcome	Booster	
					*N*	M/F	Mean Age (sd)	Time points	Instrument(s)	Unit of Measurement				Type	Number
Yates et al. [30]	2005	United States	Cardiac Rehabilitation Community Centre	n/a	64	n/a	66.7 (9.4)	Baseline, 3, and 6 months	Questionnaire	min/week	Cardiac Rehabilitation	Self-Efficacy Theory	Yes	In-person session or Phone session	1 session
Goyder et al. [82]	2014	United Kingdom	Deprived areas	n/a	282	130/ 152	54.6 (7.3)	Baseline, 3, and 12 months	Actiheart Accelerometer, and Scottish Physical Activity Questionnaire	Total energy expenditure/week, and min/week	Increasing PA	SDT	Yes	Face-to-face and telephone PA consultations	1 face-to-face and 1 telephone PA consultations
Eaton et al. [63]	2016	United States	Primary Care	n/a	210	44/ 167	48.6 (11.5)	Baseline, 12 and 24 months	7-day Physical Activity Recall Questionnaire	min/week of MVPA	Weight loss and lifestyle changes	n/a	No	Materials, exercise feedback reports, DVDs	Bi-weekly material for the first 6 months and monthly for the last 6 months. 4 reports, and 2 DVDs
Rosas et al. [76]	2020	United States	Primary Care	Overweight/ obesity	191	73/ 118	50.2 (12.3)	Baseline, 12 and 24 months	Stanford 7-Day Physical Activity Recall	min/week of MVPA	Promoting changes in diet and PA	n/a	No	Emails	Monthly emails
Anderson et al. [81]	2014	United Kingdom	n/a	Confirmed diagnosis of Adenoma	329	243/ 86	63.6 (6.8)	Baseline, 3 and 12 months	SenseWear armband	min/day being active	Promoting changes in diet and PA	n/a	No	Telephone consultations	9 phone consultations
Holt et al. [ 83]	2018	United Kingdom	Community mental health trusts	n/a	414	210/ 202	40.1 (11.4)	Baseline, 1 and 9 months	GENEActiv accelerometer	MVPA, based on 5-second epochs	Prevention of Type 2 diabetes	Self-regulatory theory, Self-efficacy, Relapse prevention model	No	Group-based booster education sessions and ono-to-one support	3 group-based sessions, and brief contact every two weeks
Allman-Farinelli et al. [86]	2016	Australia	n/a	Overweight/ obesity	248	96/ 152	27.7 (4.9)	Baseline, 3 and 9 months	International Physical Activity Questionnaire	MET min/week	Promotion of diet and PA	n/a	No	Text messages, emails, calls, and continued access to a website	Text messages and emails (every month), calls (2) and continued access to a website
Dunn et al. [113]	1999	United States	n/a	n/a	235	116/ 119	46.1 (6.6)	Baseline, 6 and 24 months	7-Day Physical Activity Recall	Energy Expenditure Kcal/kg per day	Pa programme targeting healthy behaviours	Social Cognitive Theory	Yes	Group meetings	6 months from 6–12 3 from months 12–18 2 months from 18–24
Marcus et al. [72]	2015	United States	n/a	n/a	266	n/a	40.67 (9.98)	Baseline, 6 and 12 months	7-Day Physical Activity Recall	min/week of MVPA	Benefits of PA	n/a	Yes	Printed material and booster doses	Printed material 11 times during the first 6 months, then booster doses at 8, 10, and 12 months, with a final assessment at 12 months
Valle et al. [79]	2023	United States	n/a	Cancer survivors	280	50/ 230	33.4 (4.8)	Baseline, 6 and 12 months	ActiGraph GT3X + and Godin Leisure Time Exercise Questionnaire	min/week of MVPA (both for objectively and self-reported)	PA guidelines and recommendations	Social cognitive theory, behavioural capability, self-regulation, self-efficacy, and social support	Yes	Behavioural sessions, Feedback, text messages	3 Behavioural sessions, 3 Feedback, 1 per week per 6 months text messages
Chen et al. [61]	2017	United States	Primary Care	Overweight/ obesity	40	n/a	14.9 (1.6)	Baseline, 3 and 6 months	California Health Interview Survey	Days per week	Health behaviours	Social Cognitive Theory	No	Text Message	2 per week
Keyserling et al. [68]	2008	United States	Primary Care	n/a	236	0/ 236	53.0 (7.0)	Baseline, 6 and 12 months	Actigraph Accelerometer and New Leaf Physical Activity Assessment	min/week of MPA and score on the survey	Lifestyle behaviour change	n/a	Yes	Counsellor visit Phone calls Postcards	1 counsellor visit Monthly phone calls 3 postcards
Keyserling et al. [67]	2002	United States	Primary Care	Type 2 Diabetes	200	0/ 200	59.2 (n/a)	Baseline, 6 and 12 months	Caltrac accelerometer	Kcal/day	PA, diet and diabetes care	n/a	Yes	Phone calls and group session	Monthly phone calls 1 group session
Yates et al. [85]	2017	United Kingdom	General Practices	Risk of Type 2 diabetes	808	514/ 294	63.0 (8.2)	Baseline, 12 and 36 months	ActiGraph GT3X + and International Physical Activity Questionnaire	Steps/day and MET/week	Promote PA	Self-Efficacy Theory	Yes	Group sessions	2
DeGreef et al. [90]	2010	Belgium	Hospitals	Type 2 Diabetes	41	28/ 13	n/a	Baseline, 3 and 12 months	Actigraph accelerometer and Yamax DigiWalker pedometer	min/day of MVPA	Promote PA and self-efficacy	Cognitive-behavioural therapy	Yes	Reinforcing session	1
Wyke et al. [84]	2015	United Kingdom	Professional Football clubs	Overweight/ obesity	747	474/ 0	47.1 (8.0)	Baseline, 3 and 12 months	International Physical Activity Questionnaire	MET min/week	Promote a healthy diet and PA	BCTs	No	Email and group reunion	6 emails and 1 group reunion
Latner et al. [69]	2013	United States	n/a	Overweight/ obesity	90	32/ 58	49.7 (12.3)	Baseline, 6 and 24 months	Physical Activity Questionnaire short-form	min/week of MVPA	Standard care + self-support strategies for weight loss and PA	n/a	No	Manual on behavioural strategies and skills, Meetings	1 manual weekly meetings
Vlaar et al. [89]	2017	Netherlands	Primary Care	Risk of Type 2 diabetes	314	152/ 162	44.8 (n/a)	Baseline, 6 and 24 months	Short Questionnaire to Assess Health-Enhancing Physical Activity	min/week of MVPA	Nutrition and PA promotion	Self-Efficacy Theory	Yes	Group Sessions	3–4 booster sessions
Marcus et al. [73]	2021	United States	n/a	n/a	199	0/ 199	43.8 (10.1)	Baseline, 6 and 12 months	ActiGraph GT3X + accelerometers and 7-Day Physical Activity Recall	min/week of MVPA (both for objectively and self-reported)	PA promotion	Social Cognitive Theory and TTM	Yes	Phone calls, tailored print reports, individualised report that mapped PA locations near homes. Daily messages	2 calls, and daily messages for 6 months
Risica et al. [75]	2013	United States	n/a	n/a	363	n/a	n/a	Baseline, 3 and 12 months	Godin Leisure-Time Exercise questionnaire	Total leisure time score	Weight control, nutrition, PA.	Social Active Theory	No	Phone Calls	4
Pisters et al. [88]	2010	Netherlands	Physiotherapy practices	Osteoarthritis of hip and/or knee	200	46/ 154	65.0 (8.0)	Baseline, 3 and 12 months	Short Questionnaire to Assess Health Enhancing Physical Activity	Days/week with at least 30 min of MVPA	Increase levels of activity on a daily basis.	n/a	No	Sessions with a physiotherapist	7
Kajita et al. [91]	2021	Japan	Community dwells	Risk of Dementia	49	n/a	n/a	Baseline, 3 and 36 months	Physical Activity Scale for the Elderly	Total score	Physical exercise, cognitive training, and nutrition	n/a	No	Training sessions	10 (one every 3 months)
Levy et al. [70]	2004	United States	n/a	n/a	185	59/ 126	46.8 (12.8)	Baseline, 1 and 2 months	Leisure-Time Exercise Questionnaire	MET/week	Cognitive strategies promoting a sense of autonomy, competence, and relatedness regarding exercise behaviour	Self-determination Theory	Yes	Postcard	1
Bakhoya et al. [59]	2016	United States	School	n/a	181	0/ 181	12.0 (0.7)	Baseline, 4 and 13 months	ActiGraph GT3X + accelerometers	min/hour of MVPA	PA promotion	Health Promotion Model and Self-Determination Theory	Yes	Postcard	13
Mailey et al. [71]	2014	United States	n/a	n/a	141	0/ 141	37.3 (6.7)	Baseline, 1 and 6 months	GT3X Accelerometer and Godin Leisure-Time Exercise Questionnaire	MVPA	Benefits of PA	Social Cognitive Theory	Yes	Phone calls	5
Pinto et al.* [74]	2022	United States	n/a	Breast Cancer survivors	161	0/ 161	57.3 (10.9)	Baseline, 3 and 9 months	Actigraph accelerometer and Seven Day Physical Activity Recall	min/week of bouted MVPA and min/week of MVPA	PA promotion	Social Cognitive Theory and TTM	Yes	Messages or phone calls	Weekly messages for 6 months
Salmoirago-Blotcher et al. [77]	2017	United States	n/a	History of coronary diseases	29	n/a	n/a	Baseline, 3 and 6 months	Accelerometer	min/week of MVPA	Tai-chi	n/a	No	Tai-chi classes	12 over 3 months
Fleig et al. [19]	2013	Germany	Orthopaedic rehabilitation centres	Orthopaedic or cardiac rehabilitation	884	377/ 507	49.5 (9.4)	Baseline, 1 and 12 months	Godin Leisure-Time Exercise Questionnaire	min/week of exercise	PA promotion	Self-efficacy, action planning, and perceived satisfaction	Yes	Phone calls	2
Thorsen et al. [92]	2022	Denmark	n/a	Type 2 Diabetes	214	128/ 86	59.6 (10.6)	Baseline, 3 and 12 months	Accelerometers and self-rated PA energy expenditure	min/day of MVPA and PA energy expenditure	Aerobic and resistance training	n/a	Yes	Motivational Interviews, text messages	4 interviews, 1 message per week
Kaushal et al.** [93]	2021	Canada	Rehabilitation centre	Acute coronary syndrome	13	4/9	64.2 (5.4)	Baseline, 3 and 6 months	Leisure-time exercise questionnaire	min/week of MVPA	Benefits of PA	n/a	Yes	Phone calls	3 (one each month)
Partridge et al. [86]	2016	Australia	n/a	Overweight/ obesity	248	96/ 152	n/a	Baseline, 3 and 9 months	International Physical Activity Questionnaire short form	min/week of MET	PA, vegetable consumption, take-out meal consumption	n/a	No	phone calls, text messages and email	2 calls, monthly text messages
Coleman et al. [62]	2017	United States	Hospitals	Bariatric surgery patients	51	8/ 43	49.4 (11.7)	Baseline, 6 and 12 months	Pedometer (New Lifestyles NL-800) and Behavioural Risk Factor Surveillance Survey	min/week of MVPA	An exercise programme specifically designed for this population	n/a	Yes	Group classes and counselling sessions	24 classes and 6 counselling sessions
Celano et al.** [60]	2018	United States	Medical Centre	Cardiovascular Diseases	128	52/ 76	63.1 (12.0)	Baseline, 2 and 3 months	Actigraph GT3X + accelerometer and 7-day Physical Activity Recall	min/week MVPA	Promotion of PA and health behaviours	n/a	Yes	Sessions with a motivational interview	3
Hull et al.** [65]	2018	United States	n/a	n/a	218	165/ 153	6.22 (1.07)	Baseline, 4 and 12 months	GT3X Actigraph accelerometers	% of time in MVPA	Increase PA and reduce SB Improve eating behaviours	Social Cognitive theory and food preference theory	No	Newsletter	Bi-monthly
Schultz et al. [78]	1993	United States	Diagnostic centre for cardiac PET	n/a	54	34/ 20	n/a	Baseline, 1 week and 6 weeks	Non-validated instrument	min/week of PA	Benefits of PA and injury prevention	n/a	Yes	Phone calls	2
Ko et al. [94]	2021	Hong Kong	Hospitals	COPD	136	132/4	75.0 (8.0)	Baseline, 2 and 12 months	GT3X Actigraph accelerometers	MET per day	Physiotherapy and PA sessions	n/a	No	Phone calls	15.5 (average)
Gong et al. [95]	2015	China	Community Health Centre	Hypertension	450	189/ 261	64.2 (6.0)	Baseline, 6 and 9 weeks	Self-developed questionnaire	0 = no exercise 1 = less than 15 min 2 = 15 to 29 min 3 = 30 to 59 min 4 = 1 h or more	Blood pressure risk factors and PA promotion	TTM, model of personalised medicine, and social capital theory	No	1 personal counselling and 1 group activity	2
Kattelmann et al. [66]	2019	United States	n/a	n/a	155	n/a	9/10	Baseline, 4 and 24 months	Actigraph GT3X + accelerometer and Block Kids Physical Activity Screener	min/hour of MVPA, and min/day of MVPA	Cooking, nutrition and PA promotion	Social Cognitive Theory	Yes	Monthly newsletter for lottery winners. Group events	Monthly Newsletter and 2 group events
von Ash et al. [80]	2024	United States	n/a	n/a	195	0/195	43.3 (10.3)	Baseline, 6 and 24 months	ActiGraph wGT3X-BT and 7-day Physical Activity Recall	min/week of MVPA	PA promotion	Social Cognitive Theory and TTM	Yes	Text messages and phone calls	Weekly messages for months 7 to 24
Franko et al.* [64]	2008	United States	n/a	n/a	476	204/ 268	20.1 (1.7)	Baseline, 1 and 6 months	International Physical Activity Questionnaire	min/week of MET	Nutrition and PA promotion	n/a	No	Web session	1
* Authors who were contacted to provide missing information and replied with negative responses (i.e., data no longer available) ** Authors who were contacted (including a follow-up email) and provided no reply *COPD*, Chronic Obstructive Pulmonary Disease; *MVPA*, Moderate-to-Vigorous Physical Activity; *PA*, Physical Activity; *MET*, Metabolic Equivalent; *TTM*, Transtheoretical Model															

### Risk of bias assessment and inter-rater reliability

The risk of bias was assessed using the Risk of Bias 2 (RoB2) [49] checklist. Four authors (two per article) independently assess the risk of bias for the studies included in the systematic review and meta-analysis. A traffic-light plot and a summary plot were created using the “*robvis*” R package [50], which are available in the Supplementary Material. Inter-rater reliability for the full-text screening and the risk of bias assessment among the four screeners were assessed using the Cohen’s Kappa coefficient and the following thresholds were used to assess the reliability: <0.20, slight agreement; 0.21–0.40, fair agreement; 0.41–0.60, moderate agreement, 0.61–0.80: substantial agreement; and 0.81–1.00, almost perfect agreement [51].

### Data synthesis and analysis

*Narrative synthesis.* Results from the articles that were not suitable for the inclusion in the meta-analysis because they did not report adequate data (for example, means and standard deviations regarding MVPA were not reported) were narratively summarised to describe the nature of the boosters, the number of the boosters administered, the context in which they were used, the age groups, and the target populations.

*Meta-Analysis.* We decided to include only data regarding MVPA for three reasons. First, it was the most common reported PA measure in the studies included in this systematic review, allowing us to provide a more robust meta-analysis. Second, we incorporated in the model different moderators, including baseline values. Considering that PA levels were measured using different units of measurements (e.g., daily energy expenditure, MET/hours, step count, etc.), including baseline values with such different scales (for example, a few thousands for the step count compared to a few tens for the MET/hour) would have yielded unstable results in the model. Third, including only MVPA data provides a more understandable estimation regarding PA levels. Two changes in daily MVPA (self-reported, device-assessed, or both) in minutes per day were calculated: (1) from baseline to post-intervention; and (2) from baseline to the last available follow-up. The standard error of the changes was also calculated using the raw data reported in the studies. Considering that PA has a log-normal distribution [52], the extracted mean percentage changes in MVPA min/day and their standard errors were expressed as factors of baseline mean PA and then log-transformed (e.g., for mean changes: 100 × log[1 + change in PA/baseline PA]). Back-transformed meta-analysed means, results of moderation analyses, and random-effects solutions are, therefore, expressed in percentages. The meta-analytic model was specified by including both percentage changes in the daily MVPA from baseline to the last available follow-up (t_0_ – t_2_) and from baseline to post-intervention (t_0_ – t_1_), with the predictors being baseline values of MVPA, type of measurement (device-assessed or self-reported PA), and group (control, intervention only, intervention plus booster). Data from studies that reported MPVA in min/week or min/hour was converted into min/day (Supplementary Table S11). The mixed-model procedure in version 9.4 of SAS OnDemand for Academics [53] was employed to perform a random-effects meta-analysis.

The magnitudes of the percentage changes in MVPA were evaluated using Cohen’s *d* thresholds [54]. To derive the percentage change equivalents of Cohen’s thresholds for small (0.2 standardized units), moderate (0.5), and large (0.8) changes, we multiplied the relevant Cohen’s *d* threshold value by the mean of observed baseline standard deviations for each study estimate included in the meta-analysis [55]. The resulting thresholds for small, moderate, and large effects were: 7%, 18%, and 31% for increases in MVPA, and − 7%, −15%, and − 24% for decreases in MVPA.

Rather than solely relying on null hypothesis significant testing—whereby meta-analysed effects are interpreted in a dichotomous manner as statistically significant, or not, based on an arbitrary *p* value thresholds of 0.05—we used a precision-of-estimate approach. This approach requires a more nuanced evaluation of the level of evidence for a substantial (i.e., small, moderate, and large) or trivial magnitude using the full range of values contained within the confidence interval (CI) derived from the data (given the model). Sampling uncertainty was expressed as 95% CI. We inferred the magnitude of effects in the meta-analyses by interpreting the lower and upper 95%CI and the areas of the sampling distribution, which are consistent with an alpha of 0.05. The magnitude of the effects was interpreted as follows: if a 95% CI spanned both substantial positive and substantial negative values (i.e., values less than − 7% and greater than + 7%), the sampling uncertainty for the effect was considered inconclusive; otherwise, sampling uncertainty was deemed to be conclusive and interpreted (as weak, some, good, very good, or strong conclusive evidence) based on how much area of the sampling distribution fell into substantial (i.e., small, moderate, and large) and trivial magnitudes (i.e., the area spanning − 7% to 7%) [56, 57]. Thresholds for interpreting sampling uncertainty and deciding whether an effect had weak, some, good, very good, or strong conclusive evidence are reported in Table S1.

Separate analyses were conducted for each of these three moderators: the effects of duration of follow-up (time elapsed between post-intervention and the last available PA measurement), the total number of boosters administered, and booster type (in-person, remote, or mixed). Duration of follow-up and total number of boosters administered were treated as linear numeric variables; booster type was treated as a nominal variable. In each case the moderation analysis pertained to the overall treatment effect, which is the percentage changes in the daily MVPA from baseline to the last available follow-up (t_0_ – t_2_).

### Certainty of evidence

Certainty of evidence was assessed using the Grading of Recommendations Assessment, Development and Evaluation (GRADE) approach [58]. Two authors independently applied the GRADE procedure to the meta-analysed outcome. The domains that were judged were in relation to the methodological flaws of the studies (risk of bias), the generalisability of the findings to the target population (indirectness), the imprecision of estimate, and the risk of publication bias. The heterogeneity of results across studies (inconsistency) was not judged due to the complexity of the meta-analytic model. For the judged domains, the judgement started at “non-serious” level, and then was downgraded to “serious” if problems were identified. Disagreements were resolved through discussion or, when needed, consultation with a third reviewer. The final certainty rating (“high”, “moderate”, “low”, “very low”) was generated and summarised in Table S10.

### Deviations from the protocol

First, data were meta-analysed using a different approach. According to the original protocol, Hedges’ *g* values from t_1_ to t_2_ should have been calculated. However, considering the difficulties in standardising sample-dependent estimates, percentage changes were used as a form of standardisation. Second, given the limited amount of data available in the studies, only a subgroup analysis regarding booster type was performed (the subgroup analyses regarding the participants’ clinical condition, the setting in which the study was conducted, and booster strategies framed within behavioural change theoretical frameworks could not be performed). Third, considering the variety of boosters used in the studies, a subgroup analysis regarding the differences between interventions that used a single booster compared to those that used multiple similar boosters was not feasible. Instead, this analysis was performed in a moderation analysis aiming to examine the differences between studies below (−1 standard deviation) and above (+ 1 standard deviation) the mean number of boosters administered. Finally, given that the follow-up booster durations were highly heterogenous, we decided to include the follow-up duration as a moderator and explore its impact on the booster effect.

## Results

### Study selection

**Fig. 1:**
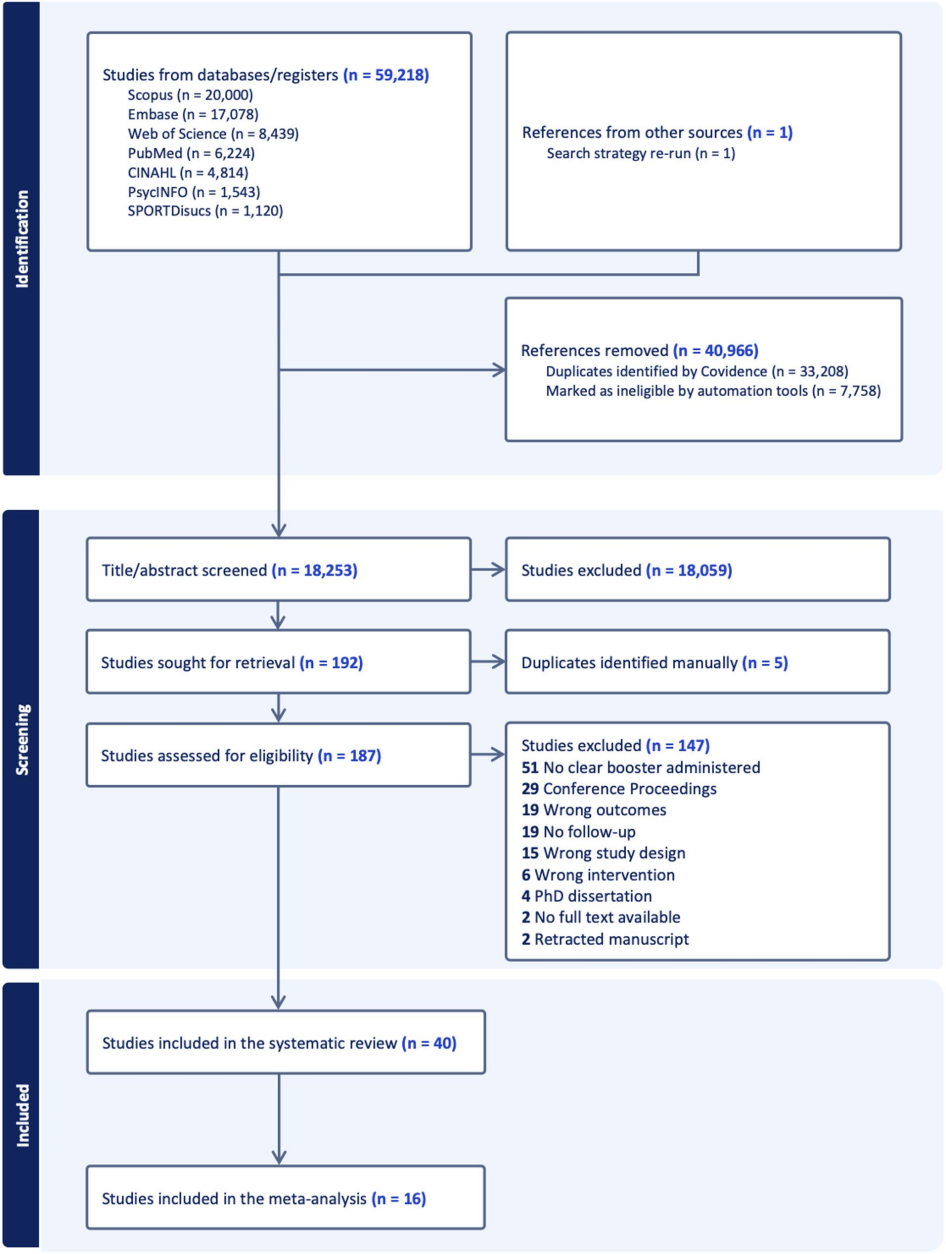
PRISMA flow diagram

The results of the search strategy are displayed in Fig. 1. A total of 40 studies were included in the systematic review, of which 16 provided data for the meta-analysis.

### Study characteristics and narrative synthesis

As seen in Table 1, almost two-thirds of the studies were conducted in the United States (*n* = 24, 60%) [113, 30, 59–80], followed by the United Kingdom (*n* = 5, 12.5%) [81–85]. Two studies were conducted in Australia [86, 87] and in the Netherlands [88, 89]. One study was conducted in each of the following countries: Belgium [90], Japan [91], Germany [19], Denmark [92], Canada [93], Hong Kong [94], and China [95]. Most studies sampled adult populations, except for two studies which included adolescents [59, 61], and two studies which investigated children [65, 66].

Study settings were varied, with the majority (*n* = 18) not conducted in a specific or clearly defined setting. However, eight were implemented in primary care or general practices [60, 61, 63, 67, 68, 76, 85, 89], three in hospitals [62, 90, 94], three in cardiac or orthopaedic rehabilitation centres [19, 30, 93], and three in community centres [83, 91, 95]. One study was conducted in each of the following settings: school [59], professional football club [84], physiotherapy practice [88], diagnostic centre [78], and a deprived community in the United Kingdom [82].

Finally, regarding the clinical condition of the population, 18 studies sampled from the general population, six studies included overweight/obese participants [61, 69, 76, 84, 86, 87], five included participants with or at risk of type 2 diabetes [67, 85, 89, 90, 92], four included participants with cardiovascular disease [60, 77, 93, 95], and two studies sampled cancer survivors [74, 79]. Finally, single studies of participants with each of the following conditions were identified: adenoma [81], osteoarthritis of hip and/or knee [88], risk of dementia [91], chronic obstructive pulmonary disease [94], and patients undergoing bariatric surgery [62].

*Type of boosters.* Table 1 shows the type and the number of boosters used in the included studies. A diverse range of boosters, as well as varying combinations of them, were employed across the studies. Phone calls were the most common type of booster. Twelve studies [19, 30, 67, 74, 75, 78, 81, 82, 86, 87, 93, 94] used phone calls – alone or in combination with other types – to support PA maintenance. The primary purpose of phone calls was to provide a form of consultation (e.g., progress with the intervention plans, support) to the participants and a reminder to adhere to the intervention. Four studies employed text messages to support participants’ engagement in PA [61, 74, 86, 87]. In-person one-to-one sessions were used in seven studies [30, 60, 82, 83, 88, 91, 95]. These sessions included intervention-specific training activities [91], motivational interviews consultations [60, 82], goal setting strategies [30], educational support related to the intervention contents [83], sessions with specialised personnel to support PA [88], and counselling sessions [95]. Group sessions were also widely used across the studies. Six studies [6, 67, 83–85, 95] used in-person group sessions to deliver either a mini-intervention or to perform a group activity aimed at reinforcing intervention contents, whereas one study used a web-based group session to support participants PA [64]. Emails were used in fewer studies. Three studies [84, 86, 87] used emails to remind and motivate participants to adhere to the intervention objectives. Two studies used printed materials as a form of booster [65, 70]. Specifically, [70] Levy et al. (2004) employed the use of postcards to remind participants about the focal points of the intervention, whereas [65] Hull et al. (2018)used a bi-monthly newsletter to reinforce intervention contents. Finally, one study used a website specifically developed for the intervention (in combination with text messages, phone calls, and emails) to support participants engagement in PA [86].

*Number of boosters*. The total number of boosters administered varied substantially across the studies, from one to 170 boosters (see Table 1). For example, [80] von Ash et al. (2024) implemented weekly text messages for 17 months, [61] Chen et al. (2017) sent two text messages per week for 3 months, and [73] Marcus et al. (2021) contacted the participants with 2 calls, and daily messages for 6 months.

### Risk of bias and inter-rater reliability

Figure S1 and S2 present the results of the risk of bias assessment for the 40 studies included in this review. Figure S1 indicates that most of the included articles demonstrated no or minimal evidence of potential bias, with the exception of three studies, which reported high risk of bias in the domain ‘bias of measurement of the outcome’ [30, 88], and in the domains ‘bias due to deviations from the intended intervention’ and ‘bias in measurement of the outcome’ [78]. Regarding the RoB2 domains, Figure S2 illustrates that most studies had a low risk of bias related to deviations from the intended interventions and outcome measurement.

Considering that the title/abstract screening was conducted in ASReview, inter-rater reliability was not calculated for this screening step. For the full text screening process and the risk of bias assessment the, the average Cohen’s Kappa values between all pairs of raters were 0.47 (moderate agreement) and 0.35 (fair agreement) respectively, whereas the average Random Agreement Probability values between all pairs of raters were 0.66 for the full-text screening process and 0.57 for the risk of bias assessment (Tables S2 and S3). This suggests that that some of the matching ratings could have occurred simply by chance—because most decisions landed in the same category of vote—so the chance-adjusted kappa values are lower even though the raters often agreed.

### Meta-analysis

**Fig. 2:**
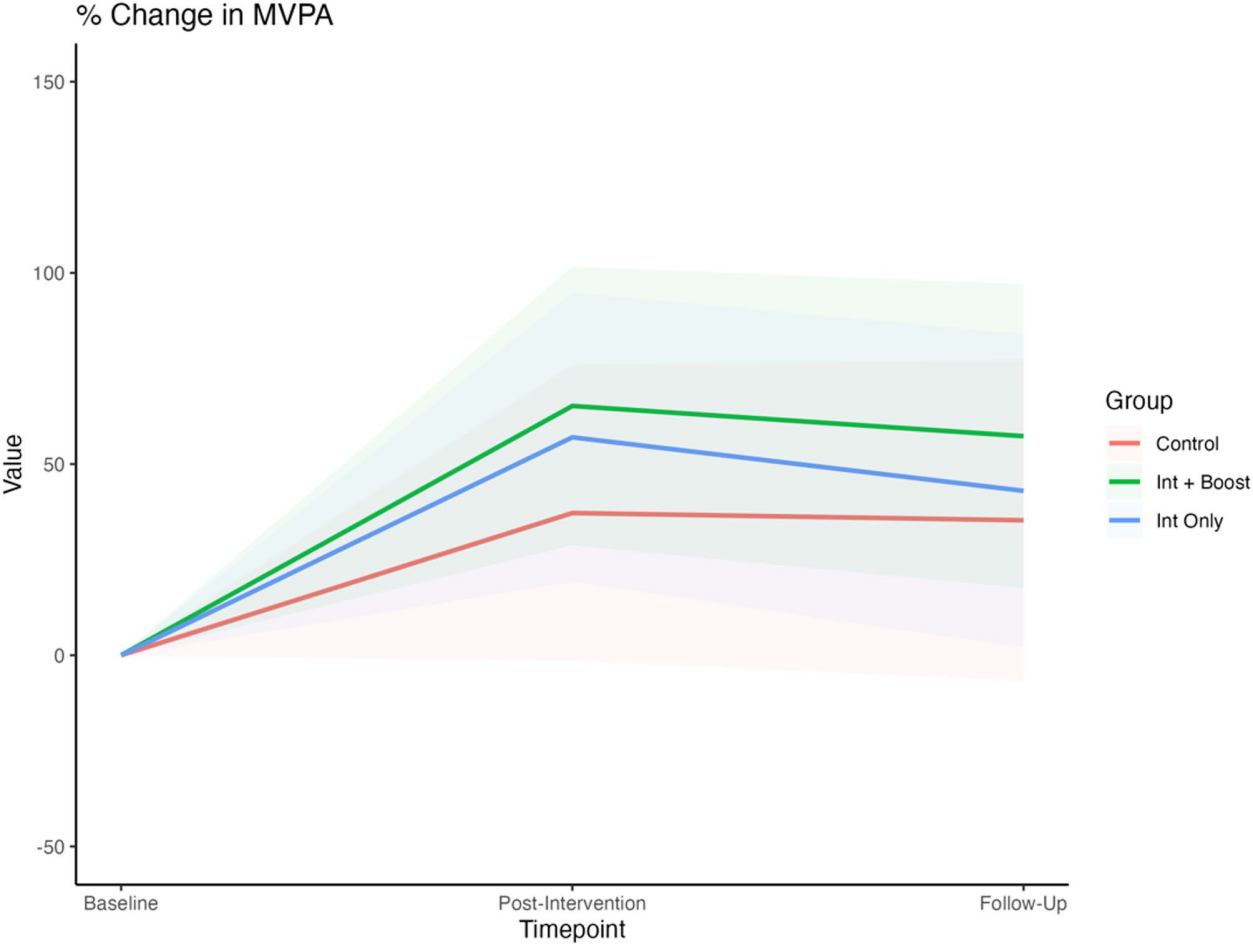
Meta-analysis of percentage changes in the daily MVPA (min/day) for the combined measures (device-based and self-reported) at three different timepoints (baseline, post-intervention, and last available follow-up) for the intervention only, intervention plus booster, and control groups. The lines represent the percentage changes in MVPA, and the coloured areas represent the 95% CI limits

Sixteen studies reported data for inclusion in the meta-analysis [59, 62, 63, 66, 68, 69, 71–73, 76, 77, 79, 80, 89, 90, 92], resulting in a total of 84 sample estimates included in the model. Six studies assessed PA with both self-reported and device-based measures [66, 68, 71, 73, 79, 80]. Figure 2 displays the percentage changes in daily MVPA (minutes per day) relative to baseline for samples in the control, intervention-only, and intervention-plus-booster conditions, averaged across device-assessed and self-reported measures. Relative to the control group, there was good conclusive evidence of increased MVPA from baseline to the last available follow-up (t_0_ – t_2_) in the intervention-plus-booster condition (Δ = 22.0%; 95% CI: − 0.1% to 44.1%; *p* = 0.051). In contrast, the evidence for change in the intervention-only condition was inconclusive (Δ = 7.7%; 95% CI: − 22.3% to 37.8%; *p* = 0.569). Post-intervention MVPA sustainment was on average 6% higher in the intervention-plus-booster group, compared to the intervention-only group. However, the 95% CI indicated only some conclusive evidence of a difference between these two groups (Δ = 6.0%; 95% CI: − 4.2% to 16.3%; *p* = 0.230). More information about the MVPA changes across the different timepoints is provided in Table S4.

**Fig. 3:**
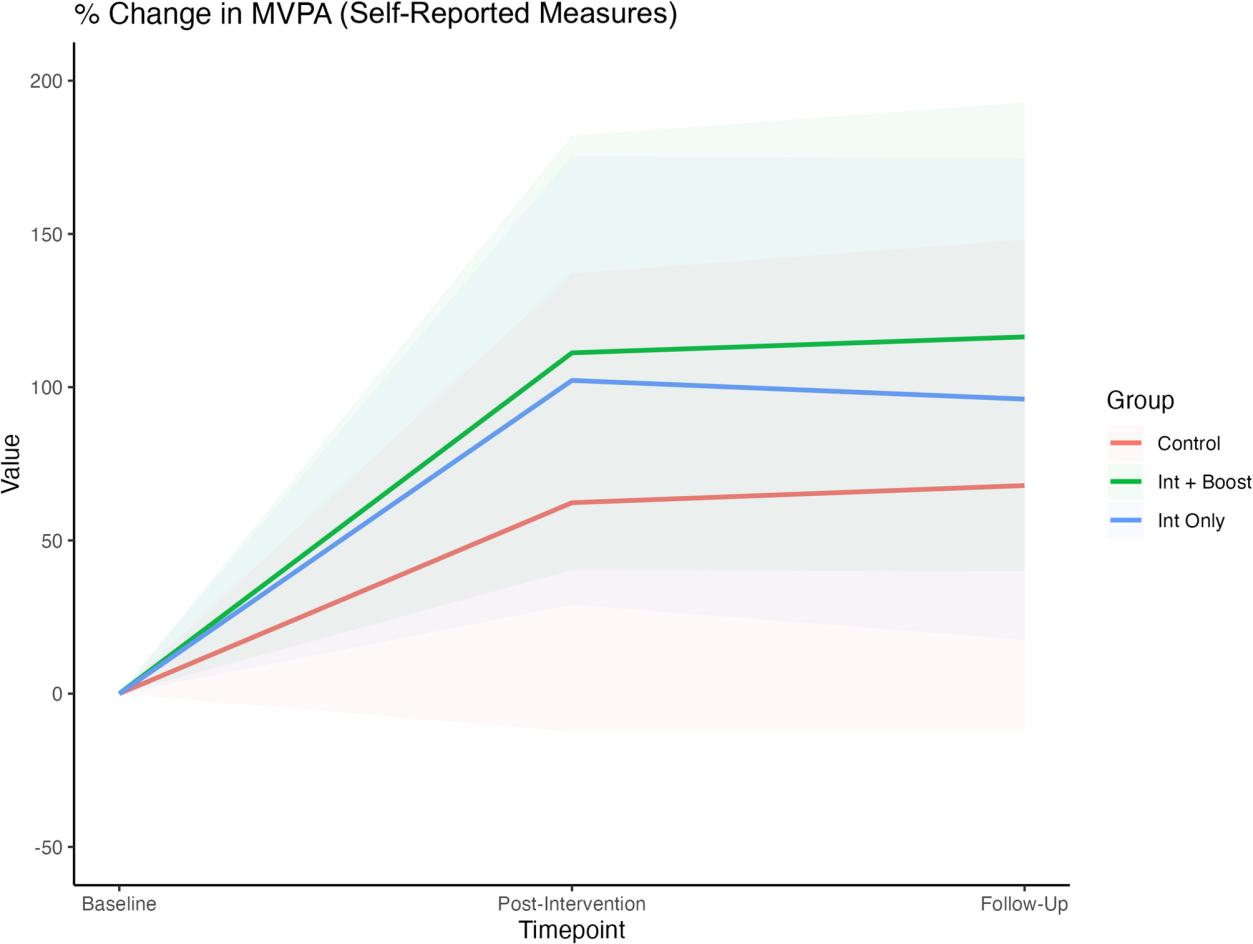
Meta-analysis of percentage changes in the daily MVPA (min/day) for the self-reported measures at three different timepoints (baseline, post-intervention, and last available follow-up) for the intervention only, intervention plus booster, and control groups. The lines represent the percentage changes in MVPA, and the coloured areas represent the 95% CI limits

Eleven studies assessed PA with self-reported measures [63, 66, 68, 69, 71–73, 76, 79, 80, 89]. Figure 3 shows the results of the meta-analysis for the daily MVPA percentage changes in the groups from such studies. Relative to the control group, there was very good conclusive evidence of increased MVPA from baseline to the last available follow-up (t_0_ – t_2_) in the intervention-plus-booster condition (Δ = 48.5%; 95% CI: 4.6% to 92.4%; *p* = 0.035). In contrast, the evidence for change in the intervention-only condition was inconclusive (Δ = 28.5%; 95% CI: − 31.3% to 87.8%; *p* = 0.300). Post-intervention MVPA sustainment was 11.3% higher in the intervention-plus-booster group compared to the intervention-only group. However, the 95% CI for this estimate indicated some conclusive evidence, reflecting uncertainty in the ‘pure’ effect of boosters (Δ = 11.3%; 95% CI: − 4.7% to 27.2%; *p* = 0.137). More information about the MVPA changes across the different timepoints is provided in Table S5.

**Fig. 4:**
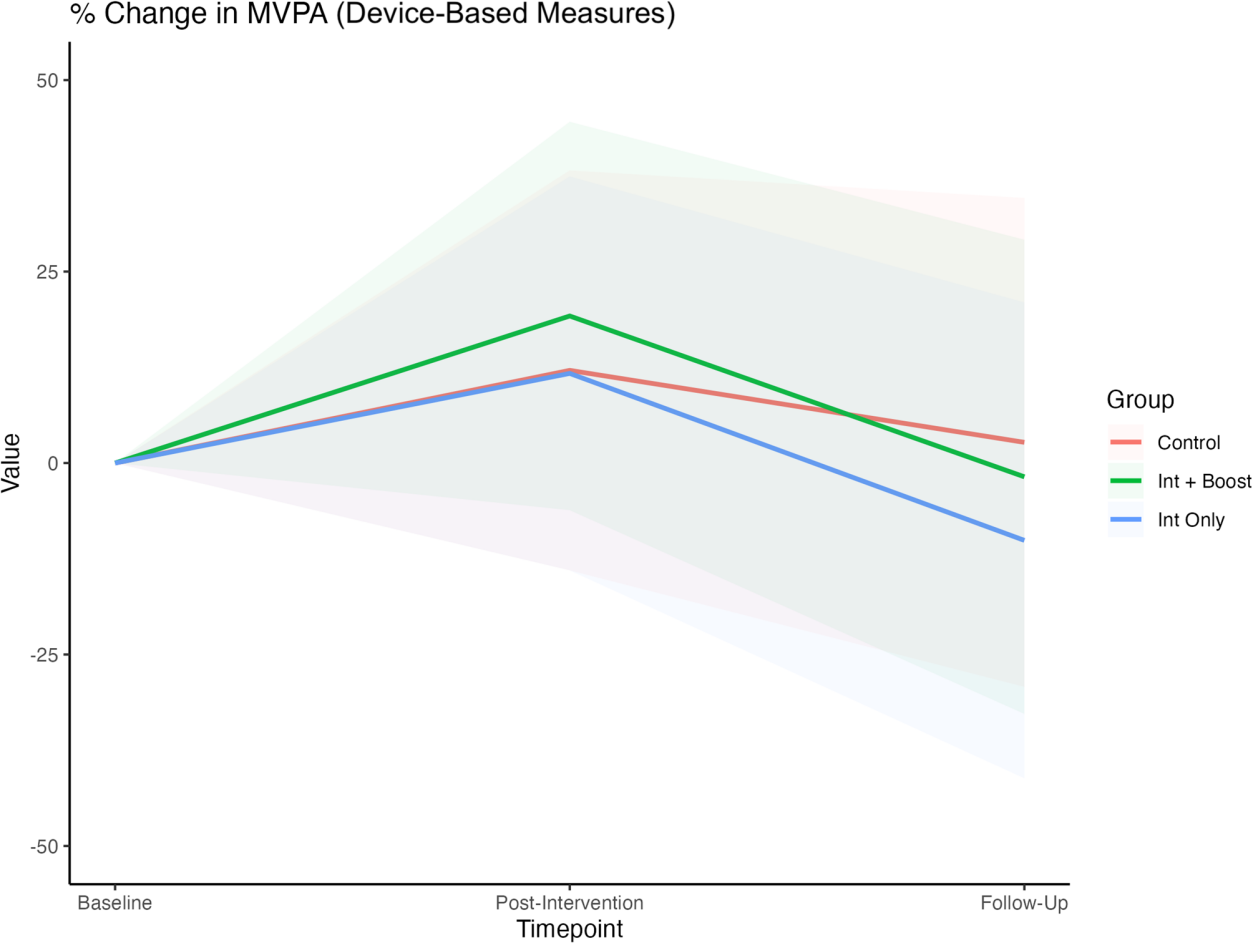
Meta-analysis of percentage changes in the daily MVPA (min/day) for the device-based measures at three different timepoints (baseline, post-intervention, and last available follow-up) for the intervention only, intervention plus booster, and control groups. The lines represent the percentage changes in MVPA, and the coloured areas represent the 95% CI limits

Results regarding studies that assessed PA with device-based measures differed from the results reported in studies that used self-reported measures to assess PA. PA was assessed with device-based measures in eleven studies [59, 62, 66, 68, 71, 73, 77, 79, 80, 90, 92]. Relative to the control group, there was good conclusive evidence of decreased MVPA from baseline to the last available follow-up (t_0_ – t_2_) in the intervention-only condition (Δ = −12.8%; 95% CI: −4.5%; 95% CI: − 13.6% to 4.6%; *p* = 0.305). Post-intervention MVPA sustainment for the intervention-plus-booster compared to the intervention-only was, however, inconclusive (Δ = 0.8%; 95% CI: − 15.1% to 16.7%; *p* = 0.912; Fig. 4). More information about the MVPA changes across the different timepoints is provided in Table S6.

Moderation analyses were conducted to estimate the extent to which each moderator predicted the MVPA percentage changes (Tables S7, S8, and S9). The moderating effect of follow-up duration was estimated by comparing MVPA changes between studies with a follow-up period of 4 months (−1 standard deviations Δ = 18.5%; 95%CI = −10.6% to 47.6%; *p* = 0.200) and studies with a follow-up period of 14 months (+ 1 standard deviations, Δ = 24.3%; 95%CI = −0.4% to 49.0%; *p* = 0.054). Results indicated an inconclusive effect (Δ_4−month FU, 14−month FU_ = 5.8%; 95%CI = −29.7% to 41.2%; *p* = 0.735).

The moderating effect of the number of boosters administered was estimated by comparing MVPA changes between studies that employed 2 boosters (−1 standard deviations, Δ = 14.8%; 95%CI = −8.1% to 37.7%; *p* = 0.179) and studies that used 43 boosters (+ 1 standard deviations, Δ = 32.8%; 95%CI = 9.5% to 56.0%; *p* = 0.011). Results showed good conclusive evidence of increased MVPA from t_0_ to t_2_ for interventions with more boosters (Δ_43 boosters, 2 boosters_ = 18%; 95%CI = 5.0% to 31%; *p* = 0.009), suggesting that when more boosters are administered, MVPA is substantially higher at the last available follow-up.

Finally, the analysis investigating the moderating effect of different types of booster (in-person, remote, and mixed) showed inconclusive evidence for changes in MVPA for the in-person boosters (*N* = 5, Δ = 10.3%; 95%CI = −13.6% to 34.2%; *p* = 0.369); good conclusive evidence of MVPA increases for remotely-delivered boosters (*N* = 9, Δ = 22.2%; 95%CI = −0.3% to 44.8%; *p* = 0.053); and very good conclusive evidence of MVPA increases for the mixed booster types (*N* = 14, Δ = 31.4%; 95%CI = 8.4% to 54.4%; *p* = 0.013).

### Certainty of evidence

We assessed the certainty of evidence for RCT in relation to MVPA changes (Table S10). The certainty of evidence was graded as low, downgraded twice, for MVPA percentage changes. For the studies, the risk of bias was judged as non-serious. However, both the Indirectness and Imprecision were judged serious due to the different interventions delivered in different settings and the uncertainty in the effects indicated by the 95% CIs. Publication Bias was assessed by conducting Egger test and was judged as non-serious due to the Egger’s test results (Supplementary figures S3 and S4). Due to the complexity of the model, Inconsistency was not assessed.

## Discussion

The aims of this systematic review and meta-analysis were to summarise and describe the boosters that have been used in PA interventions as well as to investigate the effectiveness of such boosters in promoting participants’ PA sustainment. We hypothesised that PA interventions that employed boosters would lead to more sustained PA levels in participants, and more boosters and longer follow-up durations would be associated with better PA sustainment. Results from the meta-analysis revealed a mix of expected and surprising results.

Results of this systematic review showed that a variety of boosters have been used by researchers to promote PA sustainment. Phone calls and text messages were the most common type of booster, whereas in-person boosters were less common: seven trials delivered individual boosters and six offered group boosters. The number of boosters administered spanned from a single contact to 170 contact points.

Aligned with our hypothesis, the results from the meta-analyses showed that boosters seem to be an effective strategy to promote people’s PA behaviours, suggesting that implementing booster strategies in interventions may reduce long-term declines in MVPA levels. Participants in the intervention-plus-booster group experienced an average MVPA sustainment 6% higher than the intervention-only group (ranging from − 4.2% to 16.3%). While this percentage is modest, with only some conclusive evidence due to sampling uncertainty, it should be mentioned that MVPA has an inverse dose-response relation with all causes of mortality [96], and therefore, any small changes – any additional minute added to the daily MVPA – could be meaningful. Considering that PA sustainment is a significant challenge in the health promotion field, with only a low percentage of participants maintaining regular PA in the long term [97, 98], our results showed that using boosters may offer an effective approach to sustaining intervention gains. These findings are aligned with previous reviews that investigated the effectiveness of interventions for PA behaviour change maintenance and suggested that the use of boosters (referred in those studies as follow-up prompts) may predict the success rate of interventions in sustaining long-term PA levels [21, 99].

Another important finding from this study was that the total number of boosters administered was associated with PA sustainment. Our results showed that interventions incorporating more booster strategies are more effective at sustaining MVPA levels over time. This aligns with previous research indicating that interventions with increased participant contact through boosters may lead to greater improvements in minutes of MVPA [100] and enhanced health outcomes [101]. Practically, these findings highlight the importance of determining an optimal booster dose, balancing sufficient engagement to sustain participants’ motivation without overwhelming them. Innovative strategies, such as digital delivery methods or gamification techniques, may offer viable solutions for maintaining participant engagement and delivering boosters effectively, potentially explaining the positive outcomes observed with remote or mixed-method booster interventions. Our results showed that remotely-delivered and mixed boosters (a combination of remote and in-person boosters) are more effective in sustaining participants MVPA levels over time. Despite research in this context being scarce, a narrative review has attempted to assess the effectiveness of remotely-delivered reminders (text messages and phone calls) to improve different health behaviours. Results showed that, even though such reminders were widely accepted by participants, they did not have any impact on the health care outcomes that were measured (oral contraceptive pills, acne treatment, or lupus erythematosus treatment) [102]. Even though findings from our moderation analysis showed good and very good conclusive results in favour of the remotely-delivered and mixed booster respectively, they should be interpreted with caution. First, the number of studies that used exclusively in-person booster strategies was limited (only five studies). Second, the diversity of booster types used made it difficult to categorise boosters in a more precise way, resulting in categories that are open for discussion. However, despite these limitations, results from the moderation analysis suggest positive effects of remotely-delivered boosters, which may offer a cheap and affordable way to promote sustained PA.

Finally, it is worth noting that the apparent booster benefits depended on how MVPA was measured. Across both booster and non-booster arms, the trajectory of MVPA changes was essentially parallel; the sole divergence was a marginal between-group difference that emerged only in self-reported PA, in favour of the intervention plus booster arm. This pattern echoes earlier work showing that intervention effects often look larger when evaluated with questionnaires rather than with devices, because self-reports are vulnerable to recall error, social-desirability, and expectancy biases — effects that become stronger when participants have frequent, supportive contact with researchers, as boosters provide [42, 103–109]. Thus, the small “extra” gain we detected for boosters may reflect participants’ perceptions of being more active rather than an objective behavioural change. This could be explained by the fact that none of the sixteen included trials employed a double-blind design. Because the booster strategies promoting PA (e.g., counselling calls, reminder texts, refresher classes) are immediately apparent to both participants and the staff delivering them, blinding of either party was not feasible. This unavoidable lack of participant and provider blinding might have increased the risk of reporting bias and contributed to the larger booster effects observed in self-reported MVPA. Conversely, device-based measures offer a non-self-report yard-stick, yet they are not infallible. Research has shown that device-based measures can miss activities with little vertical acceleration (e.g., cycling or resistance training), lose data during water-based exercise or non-wear periods, and their output varies with device placement and wear-time compliance [110–112]. Taken together, these findings suggest two, non-mutually-exclusive explanations. First, extra contact may prompt participants to report they are more active even when device measured activity is unchanged, resulting in expectancy/reporting bias. Second, device-based data insensitivity to certain PA activities and wear-time issues may mask real booster-related gains.

### Implications for future research

This review highlights two important research gaps. Booster dose was rarely reported in sufficient detail, preventing dose–response analyses to be conducted and leaving the minimal effective dose unknown. Second, only a few trials incorporated both device-based monitoring and self-reported measures, resulting in difficulties in assessing the discrepancies between such methodologies.

Future research should embed dose-finding or adaptive designs that systematically vary booster frequency and timing to provide evidence-informed guidance on how, when, and what types of boosters are most likely to sustain PA. For example, future research should develop a booster-reporting checklist—covering mode, content, behaviour-change techniques, and delivery costs— which could enhance comparability, while head-to-head trials of emerging low-burden formats (e.g., automated app prompts or brief “digital booster challenges” delivered through wearables) could identify scalable solutions for sustaining PA across diverse populations (e.g., clinical vs. non-clinical population, or adults vs. children). Finally, future trials should investigate the mechanism through which PA is differently reported between self-reported and device-based measures.

### Strengths and limitations

This study has several strengths. First, this systematic review and meta-analysis provides a comprehensive synthesis of the available evidence on the role of booster strategies in promoting long-term PA sustainment. One of the key strengths of this study is its rigorous methodological approach, following the PRISMA guidelines and employing both narrative synthesis and a robust meta-analytic model, both pre-registered. The inclusion of studies from diverse populations and settings enhances the generalisability of the findings. Furthermore, the use of a mixed-effects meta-analytic approach allowed for the investigation of moderators such as booster type, total number of boosters administered, and follow-up duration, providing nuanced insights into which elements of booster interventions may be most effective. Moreover, this study is the first synthesis to isolate the pure effect of post-intervention boosters, thereby clarifying how much additional benefit boosters alone can deliver.

Notwithstanding these strengths, the study also has some limitations which should be considered in the interpretation of the findings. First, variability across studies was substantial, particularly in relation to the type, content, and number of boosters administered, which limited our ability to draw definitive conclusions about optimal booster designs. Second, only a subset of the included studies reported sufficient data to be included in the meta-analysis, potentially introducing selection bias. Third, due to the complexity of the model, testing for publication bias was not performed, which can also be considered a limitation. Additionally, the small number of studies using solely in-person boosters reduced the power of the moderation analysis to detect meaningful differences between booster types.

## Conclusions

This study provides the first comprehensive evaluation of booster strategies in promoting long-term PA maintenance. Findings suggest that while booster strategies may not increase PA levels in the long term, they appear effective in reducing the decline typically observed after interventions, particularly when delivered frequently and through mixed or remote formats. Future research should further examine the booster’s optimal design, delivery, cost-effectiveness, and long-term impact across diverse populations and settings.

## Supplementary Information

Supplementary Material 1.

## Data Availability

The datasets used and analysed during the current study are available from the corresponding author on reasonable request.

## Abbreviations

PA: Physical Activity
RCT: Randomised Controlled Trial
MVPA: Moderate-to-Vigorous Physical Activity
RoB2: Risk of Bias 2
CI: Confidence Interval
